# Adaptive Convolution Sparse Filtering Method for the Fault Diagnosis of an Engine Timing Gearbox

**DOI:** 10.3390/s24010169

**Published:** 2023-12-28

**Authors:** Shigong Fan, Yixi Cai, Zongzhen Zhang, Jinrui Wang, Yunxi Shi, Xiaohua Li

**Affiliations:** 1 School of Automotive and Traffic Engineering, Jiangsu University, Zhenjiang 212013, China; 2111804011@stmail.ujs.edu.cn (S.F.);; 2 College of Mechanical and Electronic Engineering, Shandong University of Science and Technology, Qingdao 266000, China; wangjr33@163.com

**Keywords:** convolution sparse filtering, envelope spectrum entropy, particle swarm optimization, gearbox, fault diagnosis

## Abstract

Due to the superior robustness of outlier signals and the unique advantage of not relying on a priori knowledge, Convolution Sparse Filtering (CSF) is drawing more and more attention. However, the excellent properties of CSF is limited by its inappropriate selection of the number and length of its filters. Therefore, the Adaptive Convolution Sparse Filtering (ACSF) method is proposed in this paper to implement an end-to-end health monitoring and fault diagnostic model. Firstly, a novel metric entropy–time function ($H_{e}-T$) is proposed to measure the accuracy and efficiency of signals filtered by the CSF. Then, the filtered signal with the minimum $H_{e}-T$ is detected with particle swarm optimization. Finally, the failure mode is diagnosed according to the envelope spectrum of the signal with minimum $H_{e}-T$. The effectiveness and efficiency of the ACSF is demonstrated through the experiment. The results indicate the ACSF can extract the failure characteristic of the gearbox.

## 1. Introduction

The degree of precision and automation of industrial equipment is increasing with the development of modern industrial technology [1]. Condition monitoring and fault detection of the rotating parts are key technological supports for the safe and stable operation of the gearbox throughout its entire life cycle [2]. In order to ensure the normal operation of the internal combustion engine, early fault diagnosis and health monitoring of the gearbox are essential [3]. 

The process of failure diagnosis is generally divided into three steps: collecting the vibration signal of the mechanism, extracting failure characteristics from the signal, and identifying failure type according to the fault features. The extraction of failure features is the most critical step and has been the core of the development of mechanical fault diagnosis. Fault characteristics extraction approaches are generally divided into two categories: the approaches based on signal processing and the ones based on sparse feature learning. The methods based on signal processing mainly include Empirical Mode Decomposition (EMD), Fast Kurtogram (FK), Variational Mode Decomposition (VMD), etc. EMD is a method proposed by Norden E. Huang to decompose arbitrary signals into a series of eigenmode functions [4]. Cai [5] combined EMD and high=order statistics to establish a model to reconstruct the power spectrum based on the high-order spectrum, which successfully solves the problem that vibration signal is frequently interfered with by Gaussian white noise. To solve the mode mixing issue of EMD, Zheng [6] proposed the improved uniform phase EMD method, which successfully extracted the faults of the rolling bearing and rotor. The performance of EMD is limited by the cubic spline interpolation. To improve this disadvantage, Ye [7] proposed Improved EMD (I-EMD) and proved that the proposed method has superior extraction capability of failure characteristics under the same conditions. The disadvantages of the EMD method mainly include a large amount of iterative calculation, end effect and mode aliasing. In 2006, Antoni [8] introduced Spectral Kurtosis (SK), which is sensitive to transient components, into failure diagnosis of machinery and proposed the Fast Kurtogram method, which successfully extracted fault features in a noise environment. However, the parameters of a filter cannot be selected independently. In order to solve this problem, Zhang [9] introduced a genetic algorithm into FK and successfully improved the flexibility of parameter selection of this approach. To surmount the disadvantage that kurtosis is too sensitive to the nonperiodic impact interference, Han [10] proposed a fault diagnosis approach based on a generalized nonlinear spectrum significance method and successfully extracted the fault characteristics with the impact interference. In 2014, Konstantin [11] proposed variational modal decomposition. Unlike the EMD recursive decomposition algorithm, the overall framework of VMD is based on a constrained variational subject. Therefore, VMD possesses a firm theoretical foundation. However, the penalty coefficient and quantity of components greatly impact the decomposition effect in variational modal decomposition. To overcome this shortcoming, Yi [12] combines Particle Swarm Optimization (PSO) with VMD and proposes a novel approach. Zhang [13] proposed an intelligent method based on the self-organizing characteristic map with a higher failure identification rate and VMD, which successfully extracted and clustered rolling bearing faults. Sparse Filtering (SF) is a typical example of sparse feature learning methods. Lei [14] conducted preliminary research in intelligent fault diagnosis, weak signal detection, and other fields, respectively. Jia [15] proposed a Convolution Sparse Filtering (CSF) method to enhance impact characteristics effectively. Zhang [16] proposed a general normalized sparse filtering method that employed fewer samples to obtain higher diagnostic accuracy. Bao [17] introduced the Wasserstein distance into the SF method and proposed an enhanced SF method based on the maximum classifier discrepancy, which can successfully distinguish the operating conditions under the condition of speed fluctuation. Ji [18] proposed an approach based on parallel SF and achieved the extraction of sparse features from acoustic signals. Shi [19] proposed a novel SF method based on a generalized matrix norm to successfully extract the fault characteristic from acoustic signals. Han [20] proposed a fast general normalized CSF via the **L_1_-L_2_** mixed norm, which enhanced the efficiency and robustness of diagnosis. Cheng [21] proposed an unsupervised spark feature learning method that can accurately identify single faults and composite faults. Zhang [22] proposed the SF with the local structure preserved, which successfully solved the problem of the standard SF method ignoring the local structure of the input samples. 

Compared with EMD, FK, and VMD, the CSF shows superior performance in terms of excellent robustness to the signal of outliers and independence from prior knowledge. However, the superior property of the CSF is seriously affected by the unsuitable length of filter *L* and the number of filters $N_{f}$. The diagnosis accuracy and health monitoring effectiveness of the CSF is limited by the inappropriate filter length. Although the result of feature extraction becomes more accurate with the increase of the number of filters $N_{f}$, the calculation time also increases. Therefore, it is essential to determine the appropriate [*L*, $N_{f}$] for the CSF to extract the fault features efficiently and accurately. To address the issue of the parameter selection of CSF and further expand its application in the extraction of the bearing failure feature, the parameter Adaptive Convolution Sparse Filtering (ACSF) method is proposed. Firstly, the novel metric entropy–time function ($H_{e}-T$) is proposed to measure the signal filtered by the CSF. Compared to the existing metrics, the proposed metric considers both the accuracy and efficiency of fault extraction. Then, the PSO with superior global optimization performance is used to detect the filtered signal with the minimum $H_{e}-T$ value. Finally, the bearing fault is diagnosed according to the envelope spectrum of the signal with minimum $H_{e}-T$.

The rest of this paper is organized as follows. Section 2 reviews the theoretical background of the CSF approach and PSO algorithm. The $H_{e}-T$ and adaptive convolution sparse filtering proposed in this study are provided in Section 3. In Section 4, ACSF is demonstrated to extract the bearing and gear fault characteristics, which is accurately compared to CSF. Finally, the paper is summarized.

## 2. Materials and Methods

### 2.1. The Convolution Sparse Filtering Method

**Figure 1:**
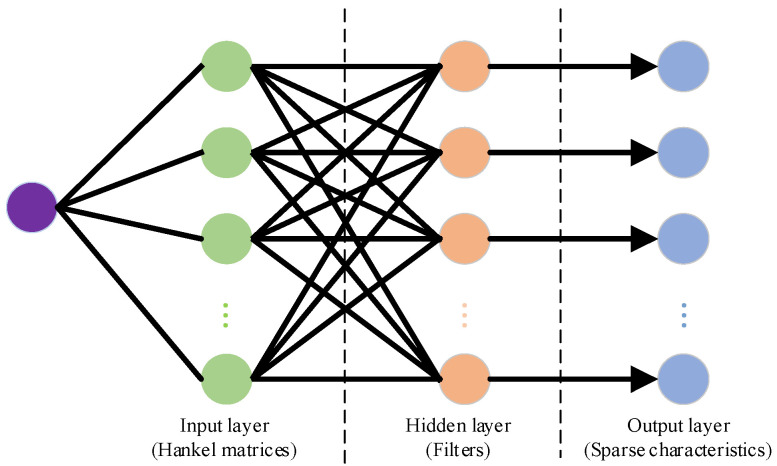
Multidimensional blind deconvolution.

Convolution sparse filtering is an enhancement of sparse filtering based on multidimensional blind deconvolution theory. Multidimensional blind deconvolution can be represented as the monolayer network, as displayed in Figure 1. The input layer is the Hankel matrix composed of the vibration signals, which are applied to activate characteristics through convolution with the hidden layer. The equation of feature extraction of the convolution sparse filtering method is as follows:

(1) f=W∗x

where f represents the extracted feature, W denotes the weight matrix, x means the input sample. The W can be regarded as a filter set. Each dimension and row of W represents a filter and its quantity, respectively. The original signal x is filtered by W and then arranged sequentially by row vectors to form the feature matrix f, which means that each row of f is the result of the filtering of signal x.

Next, the activated features are normalized and divided by the $l_{2}$ norm corresponding to the feature in that row. Then, the sum of the $l_{1/2}$ each normalized column is used as the objective function, as shown in Equation (2).

(2) $${obj}_{CSF}\left(f\right)=\sum_{i=1}^{m}{\left\|\frac{\hat{f^{i}}}{{\left\|\hat{f^{i}}\right\|}_{2}}\right\|}_{1}$$

The L-BFGS algorithm is chosen to train the model to update the weight matrix. In practice, the convolution process is replaced by the product of the Hankel matrix and the weight matrix. The Hankel matrix mentioned above is illustrated as follows:

(3) $$H=\left[\begin{array}{cc} x_{1} & x_{2}\\ \begin{array}{c} x_{2}\\ \begin{array}{c} x_{3}\\ \begin{array}{c} \vdots \\ x_{A_{i}} \end{array} \end{array} \end{array} & \begin{array}{c} x_{3}\\ \begin{array}{c} x_{4}\\ \begin{array}{c} \vdots \\ x_{A_{i}+1} \end{array} \end{array} \end{array} \end{array}\;\begin{array}{c} x_{3}\\ x_{4}\\ \begin{array}{c} x_{5}\\ \begin{array}{c} \vdots \\ x_{A_{i}+2} \end{array} \end{array} \end{array}\;\begin{array}{c} \cdots \\ \cdots \\ \begin{array}{c} \cdots \\ \begin{array}{c} \vdots \\ \ldots \end{array} \end{array} \end{array}\;\begin{array}{c} x_{O-A_{i}+1}\\ x_{O-A_{i}+2}\\ \begin{array}{c} x_{O-A_{i}+3}\\ \begin{array}{c} \vdots \\ x_{O} \end{array} \end{array} \end{array}\;\right]$$

where $x_{j}\in R^{n}$ represents the signal of fault bearing and $A_{i}$ means the input dimension of the training matrix. At this time, the activation function becomes:

(4) f=WH

### 2.2. Particle Swarm Optimization 

PSO is an optimization approach with superior global optimization capacity. The basic principle of PSO is to seek optimal solutions through mutual collaboration and information sharing between individuals in a group.

Suppose a population *X* of *L* particles in an R-dimensional space; then, *X* can be denoted as *X* = ($X_{1},X_{2},\cdots ,X_{L}$). Where $X_{i}=\left(x_{i1},x_{i2},\cdots ,x_{iR}\right)\;$(*i* = 1, 2, ⋯, *L*) means the coordinate of the *i*th particle in the R-dimensional space, which is the potential solution to the optimization problem. In addition, $V_{i}=(v_{i1},v_{i2},\cdots ,v_{iR})$ denotes the speed of motion of the *i*th particle. $P_{best}=(p_{i1},p_{i2},\cdots ,p_{iR})$ represents the individual optimal extreme value found by the *i*th particle in the process of motion. $G_{best}=(g_{1},g_{2},\cdots ,g_{R})$ is the global optimal extremum of the population. The speed and position of each particle is updated according to Equation (5) during iteration:

(5) $$\left\{\begin{array}{c} {v_{ir}}^{k+1}=\omega {v_{ir}}^{k}+c_{1}\xi \left({P_{best}}^{k}-{x_{ir}}^{k}\right)+c_{2}\xi ({G_{best}}^{k}-{x_{ir}}^{k})\\ {x_{ir}}^{k+1}={x_{ir}}^{k}+{v_{ir}}^{k+1}\; \end{array}\right.$$

where ω is the inertia factor; *r* (*r* = 1, 2, ⋯, *R*) represents the dimensionality; *i* denotes the *i*th particle in the population; *k* means the number of current iterations; $c_{1}$ and $c_{2}$ represent the self-learning and populational learning factors, respectively; ξ is a random number between [0, 1].

The first part of Equation (5) is the memory term, which is used to inherit the particle velocity of the *k*th iteration. The second part of Equation (5) is described as the self-recognition term, which is generated by subtracting the current position from the optimal position of particle. The third part of Equation (5) is described as the populational recognition term, which is generated by subtracting the current position from the populational optimal position of the particle. The next movement of a particle is resolved by the individual optimal position and the population optimal position, which represents the knowledge sharing and collaboration between the particles. PSO is applied to select the length and the number of filters of the CSF method in parallel to achieve adaptive selection of parameters.

## 3. The Proposed Method

### 3.1. Entropy–Time Function

Shannon entropy is applied to express the degree of chaos in the system due to uncertainty. The greater the disorder and randomness in the system, the higher the entropic value. Since gearbox vibration signals are characterized by uncertainty, non-linearity and non-smoothness, entropy is a superior index to measure gearbox signals. Sun [23] combined Shannon entropy with envelope spectrum and proposed the envelope spectral entropy to describe the distribution homogeneity of the frequency. The envelope spectral entropy $H_{e}$ of the signal x(t) can be expressed as:

(6) $$\left\{\begin{array}{c} H_{e}=-\sum_{i=1}^{N}p_{i}\ln p_{i}\\ p_{i}=\frac{HX(i)}{\sum_{j=1}^{N}HX(j)}\; \end{array}\right.$$

where HX(i) is envelope spectrum of x(t), and *N* represents the total number of sampling points.

$H_{e}$ is a superior indicator to merit the signal filtered by the CSF method. More abundant failure information is embodied by the signal with the smaller $H_{e}$. However, $H_{e}$ is employed as an objective function to optimize [*L*, $N_{f}$] would lead to overfitting problems in the selection of $N_{f}$. 

To address the overfitting of $N_{f}$, the entropy–time function ($H_{e}-T$) is proposed in this paper on the basis of $H_{e}$. The equation of $H_{e}-T$ is as follows:

(7) $$\left\{\begin{array}{c} H_{e}-T=H_{emin}+\mu *T\;\\ \mu =\frac{\lambda }{100}*(H_{emax}-H_{emin}) \end{array}\right.$$

where $H_{emin}$ represents the minimum value of $H_{e}$, $H_{emax}$ denotes the maximum value of $H_{e}$, *T* means the normalized CSF calculation time, λ is the degradation factor. The significance of the $H_{e}-T$ is that the selection of the $N_{f}$ is limited by the computational time of CSF. Due to the computational time is a secondary factor compared to the accuracy of the fault extraction, degeneracy factor λ is introduced to control the weight of T in the $H_{e}-T$. However, the proper value of λ is crucial to the performance of $H_{e}-T$. The excessive value of λ means the weight of time is large, which would reduce the accuracy of extraction. The overfitting of $N_{f}$ cannot be solved by the $H_{e}-T$ with a small degeneracy factor. Therefore, the value selection of λ is a problem worth discussing, and relevant analysis is presented in Section 4.1.

### 3.2. Adaptive Parameters Convolution Sparse Filtering Method

**Figure 2:**
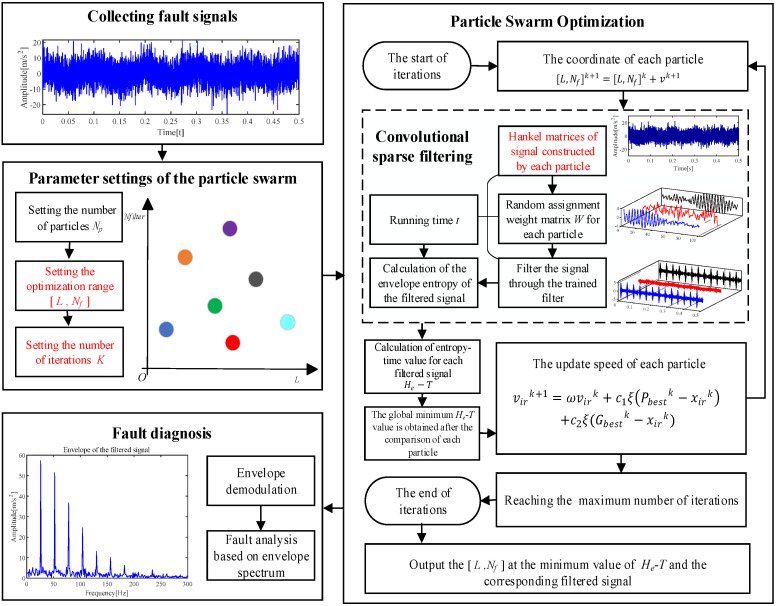
The flow of the ACSF.

Based on the above theory and analysis, the ACSF is proposed. The process of the ACSF is exhibited in Figure 2, and concise narration is as follows:

Step 1: The bearing fault signal $S_{c}\;$(1≤c≤n) is collected.

Step 2: The number of particles $N_{p}$, iterations *K* and the optimized range of [*L*, $N_{f}$] is set.

Step 3: Each particle obtains individual [*L*, $N_{f}$] and constructs corresponding Hankel matrix based on Equations (3) and (5).

Step 4: The weight matrix W of each particle is employed to filtrate the original signal. The filtrated signals are enveloped and corresponding $H_{e}$ are calculated based on Equation (6). The calculation time of Steps 3 and 4 are recorded.

Step 5: The $H_{e}-T$ of filtered signals are calculated based on Equation (7). The minimum value of $H_{e}-T$ in the population and corresponding [*L*, $N_{f}$] is the output.

Step 6: The global minimum $H_{e}-T$ is obtained by comparing the minimum $H_{e}-T$ in each iteration. The corresponding filtered signals and [*L*, $N_{f}$] are the output.

Step 7: The signal acquired in Step 6 is enveloped. The bearing failures are analyzed based on the demodulation result.

## 4. Experimental Verification

In this section, the fault feature extraction capability of the proposed ACSF is verified by the artificially implanted bearing vibration data and the abnormal noise data of the internal combustion engine gear chamber.

### 4.1. Discussion of Degradation Factor

The proposed $H_{e}-T$ is used to solve the overfitting issue for the selection of $N_{f}$. The degradation factor λ in the $H_{e}-T$ is an important parameter that determines the weight of T in the calculation result. The particle optimized results under different λ are analyzed in this section to determine an appropriate value of λ.

**Figure 3:**
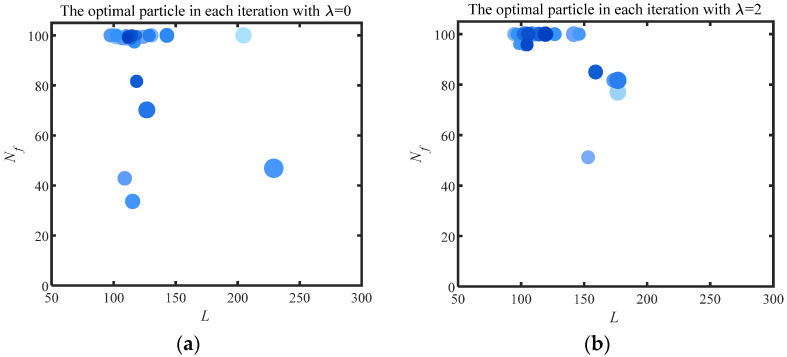

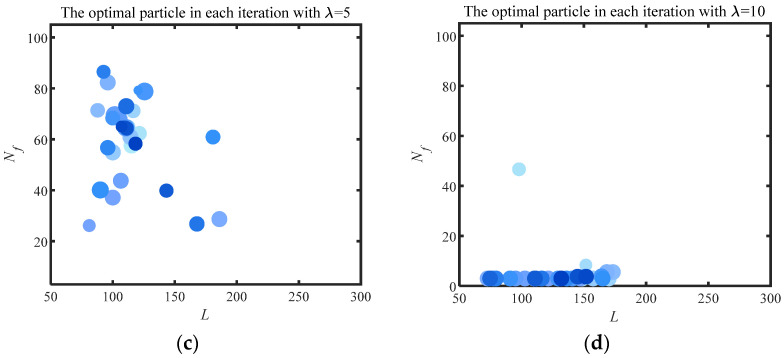
The optimal particle in each iteration with various λ.

The variable range of filter length and filter number is set as *L* = [50, 300] and $N_{f}$ = [3, 100] in this section. The optimal particles obtained from each iteration are exhibited in Figure 3, the value of the $H_{e}$ is represented by the size of the particles in this figure. Firstly, λ = 0 signifies that the objective function degenerates from the $H_{e}-T$ to the $H_{e}$, and the optimal particle positions in each iteration are shown in Figure 3a. The selection of the $N_{f}$ converges to the upper limit ($N_{f}$ = 100) in this figure. The phenomenon that the $H_{e}$ as an objective function would cause the overfitting in the selection of the $N_{f}$ is verified by Figure 3a. λ = 2 represents the weight of the *T* in the $H_{e}-T$ as 2% of the fluctuation range of the $H_{e}$. The results of λ = 2 are similar to that of λ = 0. The signal with the lowest value of $H_{e}$ is still preferentially selected by the particle due to the low weight given to *T*. The overfitting problem is not solved. λ = 5 means that 5% of $H_{e}$ accuracy is discarded by the particles for higher computational efficiency, and the optimal particles in each iteration are exhibited in Figure 3c. In contrast to Figure 3a,b, the signal with the most filters is not blindly selected by the particles. The particles clustered around 65 filters after fully comparing the results under various $N_{f}$. The results obtained by increasing λ to 10 are shown in Figure 3d. Due to the excessive weight given to *T*, the computational efficiency is more concerned with the particles than the extraction results. Most of the optimal particles are aggregated around $N_{f}$ = 3. However, fewer filters cannot accurately extract failure features, as described in Section 3.1. Therefore, λ = 5 is selected as the most appropriate degradation factor for the ACSF under comprehensive comparison. 

### 4.2. Gear Fault Feature Extraction

**Figure 4:**
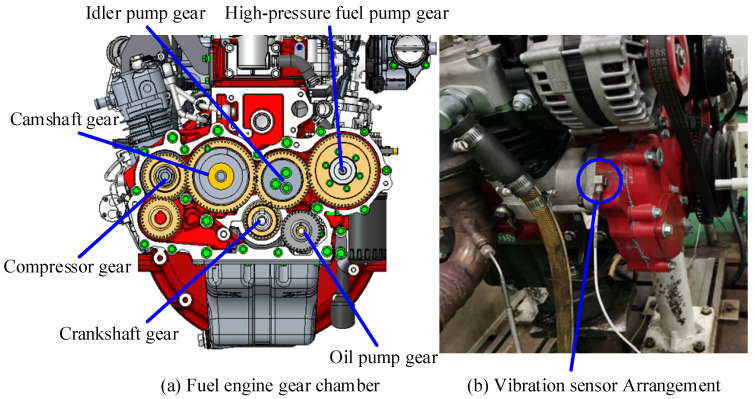
Experimental equipment.

In this section, the fault feature extraction capability of the proposed method for modulated signals is verified by the abnormal noise data from the gear chamber of a 2.5 L diesel engine. The diesel engine gear chamber profile and sensor installation position are shown in Figure 4. The crankshaft gear serves as the power input to the gear chamber. In this experiment, the crankshaft speed $f_{cg}$ = 2400 rpm, sampling frequency $f_{s}$ = 51,200 Hz, sampling time *t* = 1 s. The number of teeth of each gear in the gear room and the fault characteristic frequency at this speed are shown in Table 1.

**Figure 5:**
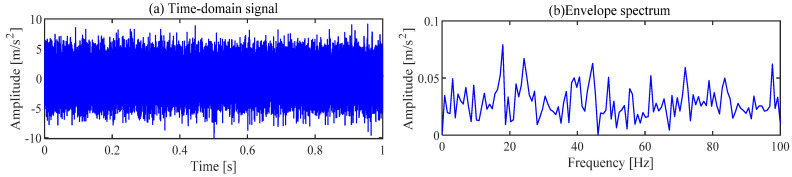
Vibration signal of the gearbox.

The gearbox vibration signal collected under the above parameters is shown in Figure 5. The periodic shocks caused by the fault cannot be observed in the time domain signal, and the fault characteristic frequency of the relevant gear cannot be observed in the envelope spectrum.

**Figure 6:**
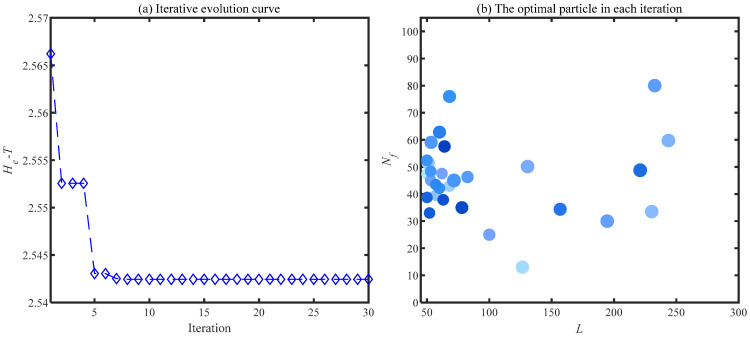
Particle swarm optimization results of gearbox signals.

**Figure 7:**
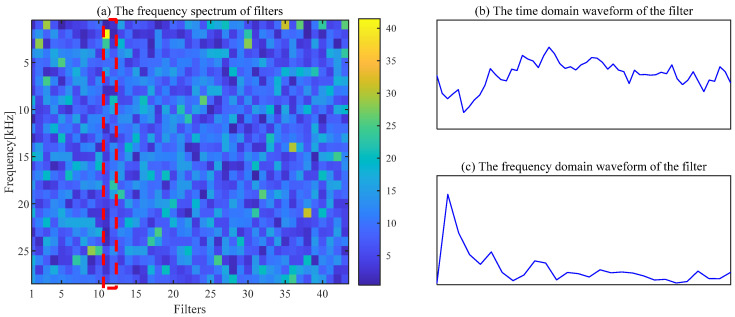
The frequency of filters and the waveform of the 11th filter.

**Figure 8:**
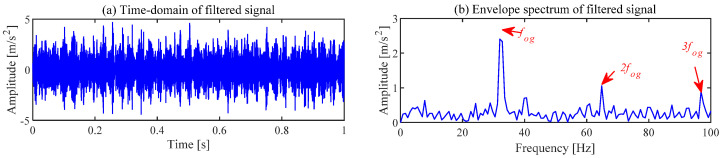
Filtered gearbox signal of ACSF.

The noise interference existing in the envelope spectrum can be effectively suppressed by the ACSF. The parameters setting of the particle swarm is the same as the Section 4.1. The optimization results of the particle swarm are shown in Figure 6. The minimum value is 2.5424 of the $H_{e}-T$ occurs at the 7th generation, and the corresponding parameter combination [*L*, $N_{f}$] is [56, 43]. The frequency spectrum of the trained filters with the above parameters is displayed in Figure 7a, and the 11th filter contains the obvious frequency contents. The signal obtained from the gear abnormal noise signal filtered by the filter is shown in Figure 8. Compared with Figure 5a, the periodic impact can be clearly observed. The fault characteristic frequency of the compressor gear (32 Hz) and its harmonics are clearly displayed in the envelope spectrum. The proposed ACSF in this paper can effectively extract the fault characteristics of the diesel engine gear chamber.

**Figure 9:**
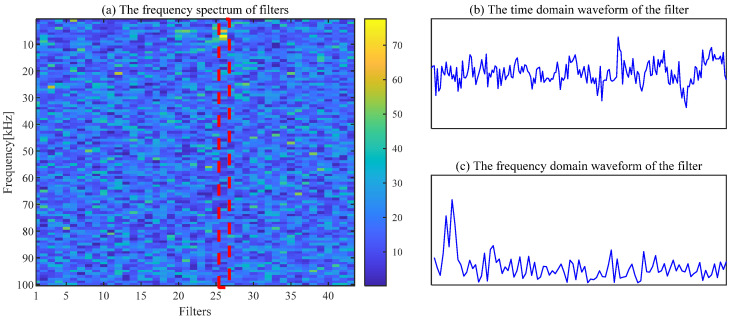
The frequency of filters and the waveform of the 26th filter.

**Figure 10:**
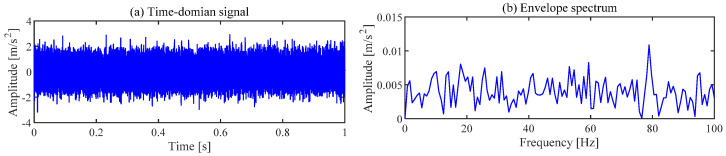
Filtered gearbox signal of CSF.

The filtering effect of CSF is seriously affected by the selection of parameters, and fault features cannot be extracted by CSF with the default parameters. The spectrum of the trained filters with [*L*, $N_{f}$] = [200, 43] is shown in Figure 9a. The waveform of the 26th filter with a significant frequency component is exhibited in Figure 9b,c. The regular shocks are not extracted in the time-domain signal, as displayed in Figure 10a. The necessity of the appropriate parameter selection for the CSF is confirmed by this phenomenon.

### 4.3. Bearing Fault Feature Extraction

In the previous section, the ability of ACSF to extract the gear signal features was validated. In this section, the bearing fault signals are used to validate the extraction performance of ACSF further. 

**Figure 11:**
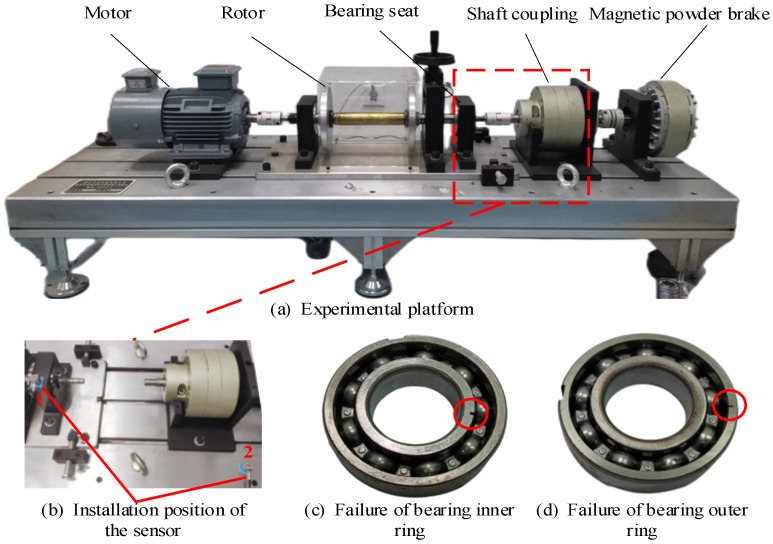
The experimental bench.

The experimental bench shown in Figure 11a is used to collect bearing fault signals to further demonstrate the superior performance of the ACSF. The experimental bench mainly includes a motor, vibration sensor, bearing pedestal, signal acquisition system and magnetic powder brake. The installation position of the sensor is displayed in Figure 11b. The sensor at position 2 is used to collect experimental signals. The bearing parameters employed for the experiments are listed in Table 2.

The bearing inner ring failure is displayed in Figure 11c. The sampling frequency $f_{s}$ = 25,600 Hz and the bearing speed *n* = 500 rpm in this experiment. The failure frequency of the inner ring $f_{i}$ is calculated by the following equation:

(8) $$f_{i}=\frac{z}{2}(1+\frac{d}{D}cos\alpha )\frac{n}{60}=45.23\;\mathrm{Hz}$$

where the quantity of rolling elements is represented by *z*, *D*, and *d*, respectively, the pitch diameters of bearing and rolling elements, the contact angle is represented by α, n represents the bearing speed.

**Figure 12:**
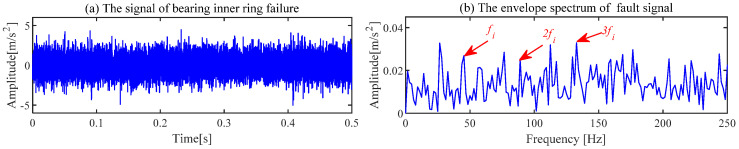
The signal of bearing inner ring failure and corresponding envelope spectrum.

The experimental signal of the fault inner ring is shown in Figure 12a. The regular shock component cannot be observed in the waveform of the time domain. The failure features are drowned out by the strong noise in Figure 12b. Bearing failure features cannot be diagnosed based on the signal of the time domain and demodulation of the original signal.

**Figure 13:**
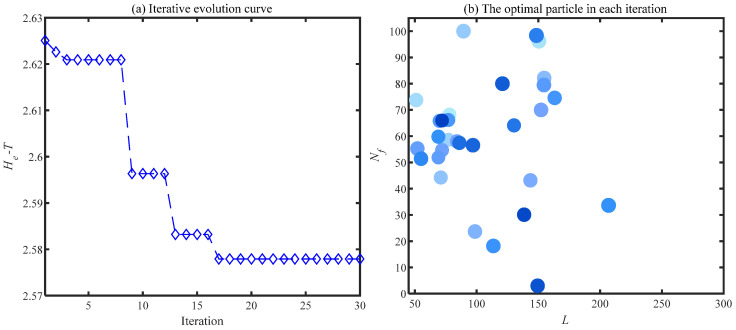
Particle swarm optimization results of bearing.

**Figure 14:**
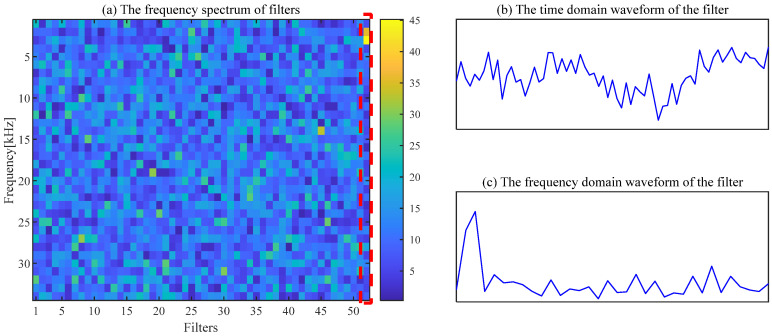
The frequency of filters and the waveform of the 52th filter.

**Figure 15:**
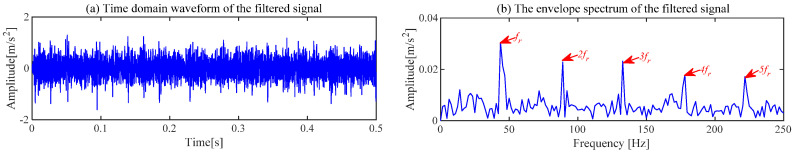
Filtered inner ring fault signal of ACSF.

The ACSF is employed to extract the fault failure of the bearing. First, the number of particles and iterations are set to *N* = 30 and *K* = 30, respectively. The optimized results of the particle swarm are exhibited in Figure 13. The minimum value is 2.5779 of the $H_{e}-T$ occurs at the 17th generation, and the corresponding optimal combination of parameters [*L*, $N_{f}$] is [69, 52]. The spectrum of the trained filters is shown in Figure 14a, where the 52nd filter has a significant frequency component. The waveforms of this filter are displayed in Figure 14b,c. This filter is employed to filtrate the original signal, and the results obtained are presented in Figure 15. The periodic shock triggered by the inner ring failure can be observed more clearly than in Figure 12a. In contrast to Figure 12b, the characteristics of the inner ring fault are noticeably exhibited in the demodulation result. The failure features of the inner ring are accurately extracted by the ACSF.

**Figure 16:**
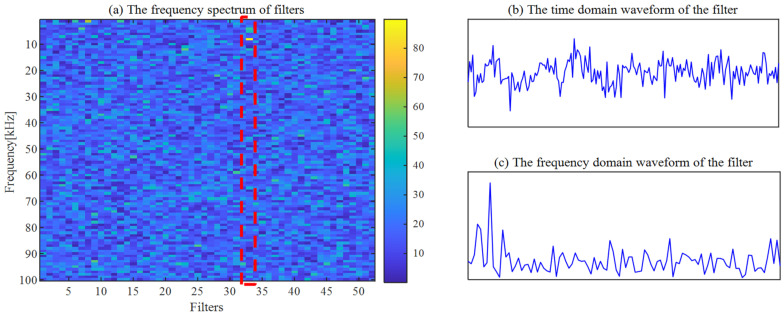
The frequency of filters and the waveform of the 34nd filter.

**Figure 17:**
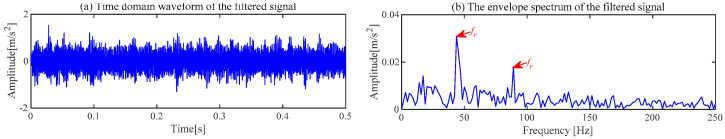
Filtered inner ring fault signal of CSF.

To demonstrate the superiority of ACSF, the CSF with default parameters is employed to extract the failure features. The spectrum of the trained filters with [*L*, $N_{f}$] = [200, 52] is presented in Figure 16a. The 33rd filter in this figure possesses a distinct frequency component, and the waveforms of this filter are exhibited in Figure 16b,c. The signal of Figure 12 is filtered by this filter, and the obtained signal is shown in Figure 15. The superior performance of shock recovery in the time domain of the ACSF is demonstrated through the comparison between Figure 15a and Figure 17a. Although the characteristic frequencies $f_{i}$ and 2$f_{i}$ can be observed, more abundant failure information is extracted by ACSF. The failure characteristics cannot be effectively extracted by the CSF with default parameters.

The fault signal from the outer race of the bearing is utilized to validate the extraction capability of ACSF further. The fault in the outer race of the bearing is illustrated in Figure 12d. The sampling frequency $f_{s}$ = 25,600 Hz and the bearing speed *n* = 900 rpm in this experiment. The fault frequency of the outer race of the bearing can be calculated by Equation (9).

(9) $$f_{o}=\frac{z}{2}(1-\frac{d}{D}cos\alpha )\frac{N}{60}=60.75\;\mathrm{Hz}$$

**Figure 18:**
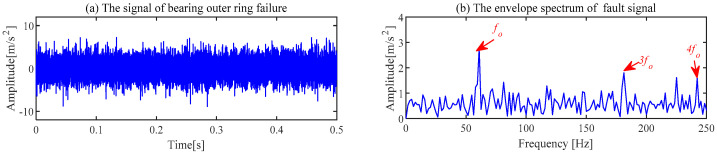
The signal of bearing outer ring failure and corresponding envelope spectrum.

The parameters in the above equation have the same meanings as those in Equation (8). The fault signal of the outer race of the bearing is displayed in Figure 18.

**Figure 19:**
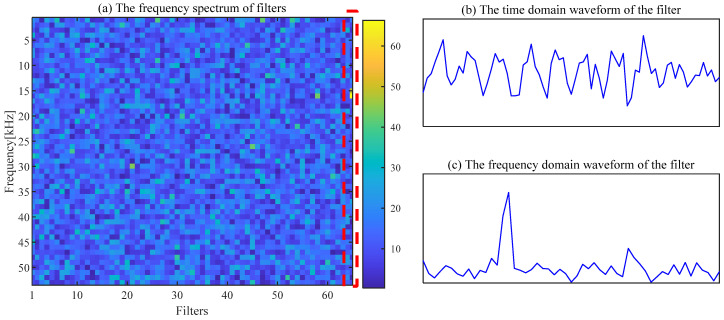
Filter spectrum diagram and the waveform of the 65th filter.

**Figure 20:**
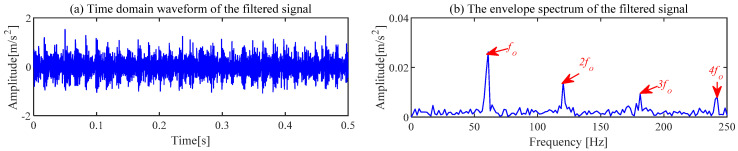
Filtered outer ring fault signal of ACSF.

The proposed ACSF method is used for the fault signal of the bearing outer ring. The minimum value of $H_{e}-T$ ($H_{e}-T$ = 2.5032) occurs in the 22nd generation, and the most appropriate [*L*, $N_{f}$] is [106, 65]. The frequency spectrum of the filters trained under this parameter is shown in Figure 19a. The 65th filter in this figure has obvious frequency components. The waveforms of the 65th filter are exhibited in Figure 19b,c. The original signal filtered by this filter is exhibited in Figure 20. Compared with Figure 18a, the noise component in the waveform of the time domain is suppressed, and the regular shocks triggered by the fault become distinct. The demodulation of the filtered signal is displayed in Figure 20b, where the frequency of failure characteristics can be clearly reflected compared to Figure 18b.

## 5. Conclusions

This paper proposed a novel method without artificial selection for end-to-end bearing fault diagnosis and health monitoring method named adaptive convolution sparse filtering. The proposed $H_{e}-T$ is the basis for selecting the filtered signal. Compared to the existing metrics, accuracy and efficiency are both considered by adjusting the degradation factor of $H_{e}-T$. The PSO is utilized to select the signal with the minimum $H_{e}-T$. The superior property of the ACSF is demonstrated by simulated analysis and experimental verification, and the optimum degradation factor λ = 5 is confirmed. Compared with the CSF with default parameters, the failure extraction results of the ACSF are more precise and distinct. *L* and $N_{f}$ have a serious impact on the filtering effect of CSF and are automatically determined by ACSF. The ACSF enhances the intelligence of the algorithm and reduces the dependence on artificial experience on the premise of ensuring extraction accuracy.

## Figures and Tables

**Table 1:** Experimental gear parameters.

Gear	Crankshaft Gear	Oil Pump Gear	Compressor Gear	Camshaft Gear	Idler Pump Gear	High-Pressure Fuel Pump Gear
Number of Teeth	30	32	37	60	47	60
Fault frequency	40 Hz	37.5 Hz	32 Hz	20 Hz	25 Hz	20 Hz

**Table 2:** The parameters of bearing.

Parameter	Bearing	Ball Number	Ball Diameter	Outer Diameter	Inner Diameter	Contact Angle
value	6205	9	7.938	52 mm	25 mm	0 deg

## Data Availability

The data supporting this study’s findings are available from the corresponding author, zhzz18@126.com, upon reasonable request.
